# Perceived unfairness in working conditions: The case of public health services in Tanzania

**DOI:** 10.1186/1472-6963-11-34

**Published:** 2011-02-12

**Authors:** Nils Gunnar Songstad, Ole Bjørn Rekdal, Deodatus Amadeus Massay, Astrid Blystad

**Affiliations:** 1 Centre for International Health, University of Bergen, P.O. Box 7804, 5020 Bergen, Norway; 2 Faculty of Health and Social Sciences, Bergen University College, P.O. Box 7030, 5020 Bergen, Norway; 3 Community Social and Economic Empowerment (COSEE), P.O. Box 137, Mbulu, Manyara, Tanzania; 4 Department of Public Health and Primary Health Care, University of Bergen, P.O. Box 7804, 5020 Bergen, Norway

## Abstract

**Background:**

The focus on the determinants of the quality of health services in low-income countries is increasing. Health workers' motivation has emerged as a topic of substantial interest in this context. The main objective of this article is to explore health workers' experience of working conditions, linked to motivation to work. Working conditions have been pointed out as a key factor in ensuring a motivated and well performing staff. The empirical focus is on rural public health services in Tanzania. The study aims to situate the results in a broader historical context in order to enhance our understanding of the health worker discourse on working conditions.

**Methods:**

The study has a qualitative study design to elicit detailed information on health workers' experience of their working conditions. The data comprise focus group discussions (FGDs) and in-depth interviews (IDIs) with administrators, clinicians and nursing staff in the public health services in a rural district in Tanzania. The study has an ethnographic backdrop based on earlier long-term fieldwork in the same part of Tanzania.

**Results:**

The article provides insights into health workers' understanding and assessment of their working conditions. An experience of unsatisfactory working conditions as well as a perceived lack of fundamental fairness dominated the FGDs and IDIs. Informants reported unfairness with reference to factors such as salary, promotion, recognition of work experience, allocation of allowances and access to training as well as to human resource management. The study also revealed that many health workers lack information or knowledge about factors that influence their working conditions.

**Conclusions:**

The article calls for attention to the importance of locating the discourse of unfairness related to working conditions in a broader historical/political context. Tanzanian history has been characterised by an ambiguous and shifting landscape of state regulation, economic reforms, decentralisation and emerging democratic sentiments. Such a historic contextualisation enhances our understanding of the strong sentiments of unfairness revealed in this study and assists us in considering potential ways forward.

## Background

In many low-income countries the health system is under serious stress due to resource constraints causing challenges in providing good quality health services. The resource constraints have a substantial influence on the qualifications of health workers and their distribution across the health facilities. The World Health Organization (WHO) in its 2006 World Health Report *Working together for health *indicates a shift from understanding poor health worker performance as caused by a lack of knowledge and skills to a focus on health workers' motivation and on management of the workforce. The WHO report states "[d]eveloping capable, motivated and supported health workers is essential for overcoming bottlenecks to achieve national and global health goals" [[1]:xv]. The 2008 *Kampala Declaration *of the Global Health Workforce Alliance points to the need "to assure adequate incentives and an enabling and safe working environment for effective retention and equitable distribution of the health workforce" [[2]:10]. These recent documents represent an increased emphasis on health worker motivation. Human resource management geared at motivating staff to provide good quality health care is vital, but there are huge challenges in managing and motivating health workers for good performance in low- and middle-income countries [[3]:668].

Working conditions are vital for health worker motivation and ultimately for the quality of the health care delivered. WHO defines working conditions as the combination of compensation, non-financial incentives and workplace safety [[1]:xvii]. A number of studies provide valuable insights into the field of health workers' working conditions, and point out diverse aspects of the relationship between working conditions and motivation to work. Motivation means to have a reason to perform a certain task. Ryan and Deci argue that "[t]o be motivated means *to be moved *to do something. A person who feels no impetus or inspiration to act is thus characterized as unmotivated, whereas someone who is energized or activated toward an end is considered motivated" [[4]:54]. The classical distinction between *intrinsic *and *extrinsic *factors of motivation emerges as important in this context, and Ryan and Deci state that intrinsic motivation "refers to doing something because it is inherently interesting or enjoyable" and extrinsic motivation "refers to doing something because it leads to a separable outcome" [[4]:55]. Extrinsic factors for motivation encompass diverse incentives and mechanisms expected to encourage a worker to increase the effort in performance of the workplace tasks. The extrinsic factors for motivation will in this context be conceptualised as 'working conditions'.

An often used definition of health worker motivation is "an individual's degree of willingness to exert and maintain an effort towards organizational goals" [[5]:1255]. In a health systems context, motivation may be perceived as the willingness to fulfil the workplace responsibilities to the benefit of the patients under the prevailing availability of resources. Studies from sub-Saharan Africa discussing health workers' experience of working conditions point to the importance of factors such as remuneration, training, supervision, recognition and transparency in human resource management [6-17]. A systematic review study of motivation and retention of health workers in developing countries concludes that financial rewards, career development, continuing education, hospital infrastructure, resource availability, hospital and management, and recognition/appreciation are core factors in low income contexts [18]. Leonard and Masatu [19] found in a study from Tanzania that working conditions alone, including salary level, cannot explain clinical performance. However, there are good reasons to believe that health workers' experience of their working conditions have a substantial impact on the level of satisfaction and motivation and further more for the effort and accuracy in their work. Hughes *et al. *argue that "[m]otivation, satisfaction, and performance seem clearly related" [[20]:369]. In this paper we address issues related to satisfaction with the working conditions. Hughes *et al. *point out that "[j]ob satisfaction deals with one's attitudes or feelings about the job itself [[20]:372]. The underlying argument of this paper is that low job satisfaction influence motivation and performance.

An important aspect of the working conditions concerns the relationship and communication between health workers and the employer. The Joint Learning Initiative, a network of global health leaders, argues that health workers "must be treated as partners in delivering health, not mere employees" [[21]:22]. Based on the work of this group, Chen *et al. *argue that the motivation of health workers encompasses "adequate remuneration, positive work and career environments and supportive health systems" [[22]:1987]. Along the same line of argument Wyss writes "[t]he commitment of health staff is determined by a number of organizational and management factors. Health workers are motivated by a feeling of responsibility and technical and financial achievement, working in an environment of mutual reliance in which differences are dealt with in a team spirit" [23].

The 2006 WHO World Health Report [1] and a range of other reports find that the performance of health workers in many low income countries is sub-optimal. Dieleman and Harnmeijer present an analytical framework for improved health worker performance [[24]:6]. This framework identifies four characteristics of the health workforce as important for performance: 1) increased availability; 2) increased productivity; 3) improved competence; and 4) improved responsiveness. We suggest an approach which takes factors at micro-level, the health facility, as a starting point in an attempt to identify factors behind perceptions of unfairness in health workers' working conditions. Such perceptions are likely to effect motivation for work and may in turn impact work performance as indicated in the literature.

The main objective of this article is to explore health workers' experience of their working conditions with an empirical focus on the rural public health services in Tanzania. The article aims to enhance our understanding of experiences of working conditions as well as of the relationship between health workers and their employers. The article calls for embedding the assessment of working conditions in a broader Tanzanian historical/political context which has been characterised by an ambiguous and shifting landscape of considerable state regulation, extensive economic reforms and decentralisation, and more recently by emerging democratic sentiments.

### The health workforce and health sector reforms in Tanzania

The public health facilities in Tanzania face a severe shortage of health workers [[25]:2, [26]:viii]. Particularly in rural areas, the staffing levels of public health facilities fall short of the staff establishment [27,28]. Many countries in sub-Saharan Africa have approached the shortage of fully-trained health workers by deploying staff with shorter training [29]. In Tanzania, two groups of substitute physicians are deployed: Clinical Officers (COs) and Assistant Medical Officers (AMOs) [[30]:1-4]. The AMOs replace the fully-trained physicians at many health facilities, while the COs are widely deployed at public health facilities and are in charge at many rural dispensaries. Variation also exists in the length of training among nursing staff in the public health sector, ranging from Enrolled Nurses to Registered Nurses and Nursing Officers at the highest level. The length of training determines the category. Medical attendants with shorter training, either pre-service or in-service, form another large and important group of staff in the public health services.

The public health sector in Tanzania has been undergoing a number of reforms with an anticipated improvement in the quality of health care. Through a decentralisation process starting with a range of parliamentary acts in 1982 [31], the district level increased its role in the planning and management of service provision. Another relevant reform is the restructuring of the salary scale of government employees. In a process that started in 2003, several cadres of health workers were grouped together in broader salary categories [[32]:177-183]. The implication is that the current salary scale provides less distinction between categories of health workers. Whereas the previous salary scale differentiated between the length and type of training, the current scale is based on fewer and broader categories of staff. Other important reforms include a new performance appraisal system implemented in 2005, while the introduction of a results-based payment scheme was planned in 2010.

Several recent Tanzania national documents emphasise the importance of a motivated health workforce. The Health Sector Strategic Plan III July 2009 - June 2015 states that the Ministry of Health and Social Welfare will ensure that "good performance is achieved and better rewarded, and that, our health workers are motivated" [[33]:ii]. Throughout this document the importance of the health workforce is emphasised. The Primary Health Services Development Programme 2007-2017, with the Swahili acronym MMAM, also has a strong focus on the health workforce [26], however with prime emphasis on how to increase the number of health workers. The Human Resource for Health Strategic Plan 2008-2013 acknowledges the challenge of maintaining a motivated health workforce and states that the current situation "leaves human resource significantly under-motivated to function effectively" [[25]:11].

At district level, the District Executive Director together with the District Human Resource Officer and District Medical Officer are responsible for implementing relevant decisions from the Ministry of Health and Social Welfare (MoHSW) and the President's Office - Public Service Management. The latter has the authority to decide on salaries and levels of allowances across all sectors.

Health workers in the public sector in Tanzania have experienced a considerable salary increase in recent years. In November 2005, junior medical doctors at Muhimbili National Hospital, Dar es Salaam, Tanzania went on strike over salary [34]. The strike was successful as it initiated a process of substantial salary increases for all health workers in Tanzania. Calculations based on government circulars on health worker salaries show a combined salary increase for junior medical doctors of more than 220% in the period from 2005 to 2008 (based on calculations for the Financial Year 2005-2006 and the Financial Year 2008-2009). The salaries of AMOs, COs and nursing staff were increased by 122-178% during the same period, with the highest salary increase for the lowest categories within each cadre [[32]:177-183, [35,36]]. Annual salary increases have been much higher than annual inflation in Tanzania [[37]:8]. In the Financial Year 2008-2009 the gross monthly starting salary of a Nurse II (the lowest category in the nursing cadre) was TSH 315,750, while the AMO salary started at TSH 435,750. Nursing staff, COs and AMOs are able to attain double the starting salary through long working experience and promotion. For example, a Nurse II could receive a salary increase of at least 28% if promoted to the next level of Nurse I [36]. Similar substantial salary increases could also apply through promotion for other cadres.

In addition to salary, health workers and other government employees are entitled to various allowances. Staff working on outreach activities, i.e. work outside the ordinary workplace, are entitled to a 'lunch allowance'. Work outside ordinary working hours is sometimes compensated by an 'extra hours allowance'. Some health workers also receive an 'on-call allowance' when being called to work outside working hours. Another important allowance is paid when attending conferences, seminars or workshops and is supposed to cover extra expenses associated with lodging and food. Health workers refer to this allowance as a 'seminar allowance'. The daily allowance for cadres such as AMOs, COs and most nursing staff can amount to TSH 65,000 per day for seminars conducted in the large cities, e.g. Dar es Salaam. In regional towns, the daily allowance is TSH 45,000, while for the district headquarters the daily allowance is TSH 35,000 [38]. The level of this allowance depends on the employee's salary level. The leadership staff at the district health service would generally qualify for a daily allowance of TSH 10,000-20,000 above the allowances listed above. The allowance system potentially adds substantial amounts to the salary.

We will argue that the salary level, financial incentives and the health sector management form an important backdrop for the discussion of health workers' motivation. This article will in particular attempt to make sense of why negative experiences related to the health workers' working conditions surface in a context characterised by increased local governance, extensive attempts at improving efficiency of the human resource management and improved financial conditions.

## Methods

### Study context

The research was carried out in Mbulu District in Manyara Region in northern Tanzania. Mbulu District is a rural district, which at the time of the last national census (2002) had a population of 237,882 [[39]:171]. The health facilities in Mbulu District comprise two hospitals (of which one is public), four rural health centres (of which two are public) and 26 dispensaries (of which 19 are public) [[40]:15]. In addition to the public district hospital located in the district centre, Mbulu, there is a large voluntary agency hospital, Haydom Lutheran Hospital, run by the Evangelic Lutheran Church of Tanzania (ELCT) some 90 km to the south-west of Mbulu.

The authors of the article have accrued work and research experience from this area in Tanzania since the early 1990s. Three of the authors (Songstad, Rekdal and Blystad) have previously carried out long-term ethnographic studies in Mbulu District and its neighbouring districts, while the fourth author (Massay) is a Tanzanian citizen with experience of health sector management and qualitative research.

### Data collection and analysis

Qualitative in-depth interviews (IDIs) and focus group discussions (FGDs) were employed. The data collection comprises three periods. During the first study period (April-May 2007) health facilities in Mbulu District were visited and initial exploratory interviews were carried out where the topics to be pursued further were identified. The bulk of the data were collected during the second study phase (January-February 2008) with more targeted follow-up interviews during the third phase (May 2009).

Six FGDs were carried out with altogether 37 participants comprising groups of AMOs, COs, nursing staff and medical attendants in the public health facilities. The FGDs were conducted with the aim of engaging health workers in a discussion of issues pertaining to their experience of working conditions. A topic guide was employed with great flexibility to allow for time to be spent on issues emerging during the discussion. The participants were articulate, and the flow of the discussions was smooth.

**Table 1:** Composition of FGDs

FGD number	Category of staff	Location	Participants	Men	Women	Average age
FGD 1	Nursing staff	Health Centre	7	0	7	age not recorded

FGD 2	Medical attendants	District Hospital	7	1	6	37

FGD 3	Medical attendants	District Hospital	5	1	4	33

FGD 4	Nursing staff	District Hospital	5	0	5	33

FGD 5	Nursing staff	District Hospital	6	0	6	38

FGD 6	Clinicians (AMO/CO)	District Hospital	6	6	0	38

A total of 33 IDIs were carried out. Twenty-eight IDIs were conducted with staff at health facilities spanning the district hospital, health centres and dispensaries. During the course of the research, complaints about the working conditions emerged as an important concern to the health workers. Hence, it was deemed important to also carry out IDIs with health sector administrators to elicit information on how the working conditions are regulated. Five IDIs were carried out with administrators responsible for human resource management in the district administration and at the district hospital.

**Table 2:** Overview of IDIs

Category of informant	Number of interviews
Assistant Medical Officers (AMO)	4

Clinical Officers (CO)	9

Nursing staff	10

Medical attendants	5

The aim of the sampling was to broaden the data material both in terms of geographical distribution and in terms of type and size of health facilities. Health facilities were selected to ensure a geographical coverage within the district. The most important facility was the district hospital and the majority of the IDIs and FGDs were carried out here. Some IDIs and one FGD were carried out at a rural health centre. In addition, several IDIs were conducted at more distant rural dispensaries in the district. All IDIs were carried out in locations providing necessary privacy to allow interviewees to speak freely.

The process of data collection through IDIs was characterised by open and explorative interviews in the early phase and by longer, more focused and more systematic interviews during the later phases. Interview guides were used during all the interviews. Of the 33 IDIs, 19 were tape-recorded and transcribed. Rapid note-taking was applied during the IDIs in the initial phase. One of the co-authors (Massay), in close collaboration with the first author, carried out the IDIs at the rural dispensaries.

In addition to the formal IDIs and FGDs, the first author engaged in informal discussions with health workers and residents living in the district centre and nearby villages related to the topic in question. These discussions proved to be a valuable source of information that was further explored. Documents collected during the course of the research were further central sources of information.

Songstad and Massay carried out the data collection using Swahili, the national language of Tanzania. All the FGDs and the recorded IDIs were translated and transcribed in English by the Massay and other assistants. The first author checked all transcripts and verified the translation from Swahili to English. The analysis of the material started with an initial review of the interview notes and audio files during the data collection phase. Notes, audio files and transcribed IDIs and FGDs were later imported into NVivo 8 for the purpose of data management. The material was subjected to a thorough review and coding of the content for identification of central themes. The codes were grouped into broader categories covering the emerging themes. A systematic search for recurring themes, patterns and contradictory or ambiguous statements was carried out by several of the authors.

### Research ethics

The present study is part of a collaborative research venture funded by the Research Council of Norway entitled *Strengthening human resources for health: A study of health worker availability and performance in Tanzania*. The National Institute of Medical Research (NIMR) in Tanzania granted ethical clearance for the project, ref. NIMR/HQ/r.8a/Vol. IX/433, dated 25 May 2006. Research permit for the research component upon which this article is based was also granted by the Tanzania Commission for Science and Technology (COSTECH). The initial research permit 2007-59-CC-2006-193 was issued 12 March 2007, with the extensions 2008-181-ER-2006-193 (30 June 2008) and 2009-250-ER-2006-193 (1 October 2009).

After obtaining the research permit, a letter of introduction from the Regional Administrative Secretary in Manyara Region was obtained. This letter was thereafter presented to the District Administrative Secretary and the District Executive Director in Mbulu District. The subsequent arrangements for interviewing health workers in the district health services were made through the office of the District Medical Officer. These steps were repeated for each renewal of the research permit at COSTECH.

The research complied with the relevant regulations of the Tanzania National Health Research Forum regarding information for the study participants [[41]:18]. All the informants participating in the IDIs and FGDs received information about the research both in writing and verbally before signing a consent form.

## Results

The IDIs and FGDs revealed substantial frustration with the working conditions among the health workers interviewed. Experience not only of unsatisfactory working conditions, but also of distress linked to a perceived lack of fundamental fairness dominated the interviews across different health facilities and across different cadres. In the following section, extracts of the main themes emerging in the discussions will be presented with specific reference to experiences related to salary level, promotion, recognition of work experience, allowances and upgrading opportunities, as well as to human resource management.

### Dissatisfaction with salary level

The large majority of the informants maintained that the salary they receive is not satisfactory. The dissatisfaction was related both to the perceived lack of parity between salary and workload as well as to the experience and frustration of the salary not covering the basic costs of living. Long working days and working outside the prescribed working hours were particularly commented upon by many. One CO explained:

We leave work at 4 pm but the work does not end there. Patients also come at night. We don't rest, and we work for many hours. How can we be satisfied with the salaries we get? (CO, dispensary, IDI)

Informants pointed out the increasing demands on performance and efficiency. Demands related to record-keeping and reporting were seen as an additional burden. One CO said:

We do much more work than what we are paid for, and we do much more work than in the past. There is much follow-up and efficiency is demanded in every field of activity. The salary we get is very small and I would dare to say that much of it is voluntary work. (CO, dispensary, IDI)

Many of the health workers interviewed, in particular medical attendants and nurses, further brought up the challenging conditions they work under in relation to the salary level. One nurse explained:

Working as a nurse is a vocation, but we have difficult working conditions and a risky working environment. You may be helping an HIV-positive woman during delivery. Your own life is at risk, and yet the payment is so small, making you unhappy and disturbed. (Nurse, hospital, FGD)

Across all cadres, the health workers interviewed argued that they face a high workload with steadily increasing expectations, and that the salary does not match the work. Many of the health workers argued that the salary increase in recent years has been counteracted by inflation. It was argued that the government has a dual responsibility in providing adequate salaries and in ensuring that the cost of consumer goods and other expenses are maintained at a level that is manageable. One medical attendant said:

The government has to consider controlling the prices of consumer goods to stop the price increases we see. Every month the prices have increased. Everything has increased. (Medical attendant, hospital, FGD)

The health workers claimed that the salary is too low to make ends meet, and that there is a need for other sources of income in addition to the salary. In particular, many experience the burden of the cost of their children's education as challenging, but also the cost of the construction of adequate housing represents a considerable expense to be covered by the salary. Sending children to school beyond the free seven years of primary education, i.e. to secondary education, was raised as a major challenge. One CO at a dispensary argued:

The salary I am getting is not even enough to meet the basic needs, and I find it difficult to pay school fees for my children. (CO, dispensary, IDI)

The salary level surfaced as an important and very fundamental concern to all the study participants regardless of cadre, and it was experienced as a paradox that professional health workers with many years of education did not earn enough to secure education beyond primary school for their children. The health workers interviewed generally expressed concern about the quality of the government-run secondary schools. Some health workers stated that they try to send their children to secondary schools considered to be of better standard. The fees for these schools can however be very high and difficult to raise for government-employed health workers.

There was also considerable frustration linked to the comparison of salary with other government employees. One nurse explained:

An employee at the district council gets a very high salary and plenty of allowances. Their work only involves paperwork, simply handling paper. While we, the nurses, caring for the souls of human beings and saving the lives of children, are paid less. This is something the government has not considered well. (Nurse, hospital, FGD)

Such comparisons influence health workers' perception of whether they receive a fair salary for the work they perform. The comparison with other government employees and their allegedly better working conditions, in particular in financial terms, was repeatedly brought up in the IDIs and the FGDs.

### Delayed promotions

Health workers are placed on the government salary scale according to their formal qualifications. There are four to five salary levels within each of the main cadres of health workers, and through promotion an employee moves upwards to the next level on the salary scale. The informants reported very specific expectations about the intervals for promotion, and referred consistently to being entitled to promotion every three years. However, many interviewees claimed to have stayed in the same position for a much longer time without promotion. One nurse explained:

In my letter of appointment I was told that I am eligible for promotion after three years of service in one position. But I do not know where the negligence is, somewhere in the Ministry or where? (Nurse, hospital, FGD)

The three-year frequency of promotion was considered a basic right, and delayed promotion was thus seen as a violation of the contract between the employer and employee and constituted a major source of dissatisfaction. The study informants also tended to compare their own promotion schemes with employees in other governmental sectors. The most frequent comparison was with teachers. One CO argued:

The teachers always get promotion every three years, but in our case there is no change in position at all. I think it is not fair. Look, you have provided a very good service to your employer, but he denies you justice by not giving you promotion. (CO, dispensary, IDI)

Another CO relayed his concerns about the impact of delayed promotion on his pension:

I am approaching retirement age, I only have four years left at work, and I will probably have a poor pension. Promotion is not handled properly, and there is no one in the district administration who cares about our promotion.

and furthermore:

If you follow up on promotion you will waste your time. You will run around in the offices without results. This is very discouraging and demoralising. (CO, dispensary, IDI)

Promotion was previously handled at central government level but following recent decentralisation reforms it has become the responsibility of the local government. The blame for the delays experienced was thus directed both at central government and at the district level administration.

### Lack of recognition of long working experience

The salary scale in use until the implementation of the new scale from 2003 onwards contained more categories to distinguish between the length and type of training. The current salary scale lumps together several of the previous categories in larger and broader categories. In addition to grouping together different types of training, the current scale offers fewer opportunities for differentiating salaries. Health workers with long working experience thus sometimes find that colleagues within the same cadre with shorter working experience but with longer formal training are placed on the same or even higher salary levels than themselves. The experience of being bypassed in salary by colleagues with shorter working experience was a cause of great dissatisfaction. One nurse complained:

I think I am not fairly paid, because right now I have 38 years of work experience, but I get the same pay as young employees. I have a very long work experience. Others are recently employed and have no work experience. I have taught them how to work but they have the same or even higher salary than I have. (Nurse, hospital, FGD)

Another nurse expressed forcefully:

These newly-employed children, even if they have been to various nursing schools, they cannot do better than me at work. (Nurse, hospital, FGD)

Among the nurses, the Registered Nurses have the longer training and complaints were also raised about the lack of differentiation between the Registered Nurses and the Enrolled Nurses with shorter training.

The lowest paid group of staff in the district health services, the medical attendants, has been subject to changes that have been experienced as dramatic. The changes to the salary scale brought health workers who previously enjoyed a professional status into the lower medical attendant cadre. This applies for example to some laboratory staff with a formal training in laboratory work. These developments initiated extensive complaints from staff in this group.

### Unfair allocation of allowances

The seminar allowance constitutes an important addition to the salary of the health workers, and many health workers interviewed expressed that they would like to attend seminars as often as possible. Senior administrators at the district hospital argued that attention is paid to fair distribution of seminar participation among employees at all levels. However, the staff expressed great dissatisfaction with the opportunities to attend seminars and with the decision making procedures related to participation in seminars. Medical attendants argued that they very rarely get an opportunity to participate in seminars, and said that seminars with the attached allowances are largely a prerogative of the higher-level staff. Some categories of nurses, in particular those who work in the general wards of the district hospital, also claim that they very infrequently get the opportunity to attend seminars.

It is well known that only a fraction of the daily seminar allowance is needed to pay for accommodation and food, thus there is an opportunity to save a substantial part of the allowance. One nurse explained the differences that seminar attendance is perceived to create:

You may work for a long time without attending a seminar whereas others attend often. Some of those who go to seminars may sometimes not even have time to come back, they move from one seminar to another. That is why some are successful in life while others are not. Their economic situation changes, they can build a house, even send their children to the university, they have got the money. (Nurse, hospital, FGD)

Staff members indicated that the unfairness experienced relating to who is chosen has implications on their motivation to work. One AMO explained:

Those who appear obedient to the management get the opportunity from the bosses to attend seminars irrespective of their work specialisation. The selection for seminars is biased and causes a lot of misunderstandings and demoralises the workers. (AMO, hospital, FGD)

The 'seminar allowance' is divided into three levels, where the majority of staff in the district health services fall into the middle bracket. Other allowances also depend on the salary scale, for example the 'on-call allowance' available to certain groups of staff such as clinicians and staff in the operating theatre, laboratory and radiology services at the district hospital. Nursing staff qualifying for this allowance consider it unfair that they receive a lower 'on-call allowance' than for example the AMOs. One nurse explained:

Concerning motivation of staff, the AMOs get TSH 10,000 in 'on-call allowance' whereas the nurses only get TSH 5,000. Is this fair? (Nurse, hospital, FGD)

The health workers interviewed claimed that sometimes the level of allowances was subject to district-specific decisions. On several occasions the affected staff complained to the District Executive Director and corrections were reported to have been made to the allowance level.

Unfairness in relation to overtime work was also a theme brought up throughout the interviews. The majority of staff members interviewed argued that they work more than ordinary working hours, but said that only a small minority have the privilege of receiving the 'extra hours allowance'. Hence, while some health workers reported receiving this allowance as compensation, others argued that they are sometimes compensated by time off. Others claimed that they carried out a lot of 'voluntary work', as informants referred to it. The lack of a uniform system for compensation for extra hours of work was perceived as unacceptable.

### Restricted access to training and upgrading

The majority of the health workers, with the exception of those close to retirement age, expressed that they would like to be upgraded through further training. The possibility of upgrading is defined by the needs of the district health services and by the availability of courses at the relevant training institutions. A recently introduced requirement of secondary school education however restricts staff with only primary school education from undertaking further training. This is a cause of much disappointment, as one nurse expressed:

I think for us who were employed those days with our basic primary education are now being treated badly by being forced to get a Form IV certificate. I think that level is not necessary.

and furthermore that:

I would like to improve and upgrade through further training in order to increase my skills and ability to work even better, but I don't think that Form IV certificates are of great importance in assisting women during labour. (Nurse, hospital, FGD)

Health workers facing this bar on further training complained strongly about the government regulations and considered it fundamentally unfair, as the requirement for secondary school education had not been part of the requirements when they entered the nursing profession and now blocks access to further training.

### Lack of transparency in human resource management

Health workers who complained about limited access to seminars, lower allowances and inadequate compensation for additional work hours generally blamed both the hospital management and the district administration for the unfairness experienced. Hence, human resource management at district level as well as at hospital level was often cited as a reason for the unfairness experienced. Problems related to the manner in which rules and regulations are implemented were also brought up as a cause of frustration, and some informants indicated that the managerial practices had a direct impact on their motivation to work. One AMO explained:

I think those responsible for our employment benefits should perform their duties and responsibilities very fairly so that we become motivated to work. (AMO, hospital, FGD)

The fundamental problem of access to information was moreover addressed by many of the informants. One AMO explained:

No one is willing to show how salary scales are categorised by government regulations or by standing orders. They keep it an official secret. (AMO, hospital, FGD)

One CO at a dispensary stated:

We don't know our salary scale or our annual increment, and whether we are getting the right payment or not. We can't claim anything when we don't know. (CO, dispensary, IDI)

The shortcomings in distribution of information caused a substantial perception of unfairness, in particular pertaining to issues concerning allowances and promotion.

Health workers' comparison of health sector salaries and the salaries of other civil servants, and their experience of unfairness, were cited as the reasons for the strike initiated by medical doctors at Muhimbili National Hospital in 2005. It was claimed by informants that the government later responded by limiting access to information about salary scales and other relevant information on working conditions so as to restrict such comparisons.

### Varying experience of unfairness

Government employees in Tanzania have experienced dramatic shifts in their working conditions during the last decades. A number of attempts at improvements in salary levels, human resource management and staffing levels, as well as in the availability of equipment and drugs, have taken place. There was however variation among the health workers interviewed regarding their recognition of the impact on working conditions. Some of the health workers saw little improvement and expressed great dissatisfaction, whereas others acknowledged some improvements. The study also found a range of examples of health workers demonstrating limited knowledge about the relevant rules and regulations which influence working conditions.

Some important lines can be drawn between the replies from staff at the lower and the upper ends of the health worker hierarchy regarding dissatisfaction with working conditions. Medical attendants expressed a deep dissatisfaction relating to salary, access to allowances and very few opportunities for upgrading through training. The nursing staff expressed largely similar complaints but with more emphasis on access to allowances. One explanation may be that there is great variation within the nursing staff cadre regarding access to allowances, particularly related to the seminar allowance. Moreover, nursing staff who fulfil the educational requirements have greater opportunities for further training than medical attendants. The COs and AMOs also emphasised issues pertaining to salary allowances, but more frequently than the other groups pointed to the lack of transparency in human resource management.

Moreover, long working experience with lengthy exposure to perceived unsatisfactory working conditions seems to increase the level of dissatisfaction. This may be linked to delays in promotion with the attached salary increases. Another reason for dissatisfaction among health workers with long working experience is that many only had primary school education prior to their training which limits their current opportunities for further training and upgrading. Another pattern found in the material is that health workers at the dispensaries, the smallest health care unit, expressed greater motivation to work, more support for district level management and greater acknowledgement of the recent improvements than staff at the more centrally located units.

It should be added that most rural districts in Tanzania, including Mbulu District, consist of multiple ethnic groups. A striking feature in our material is that we found no references to perceived unfairness due to favouritism or discrimination based on ethnic affiliation.

## Discussion

**Figure 1:**
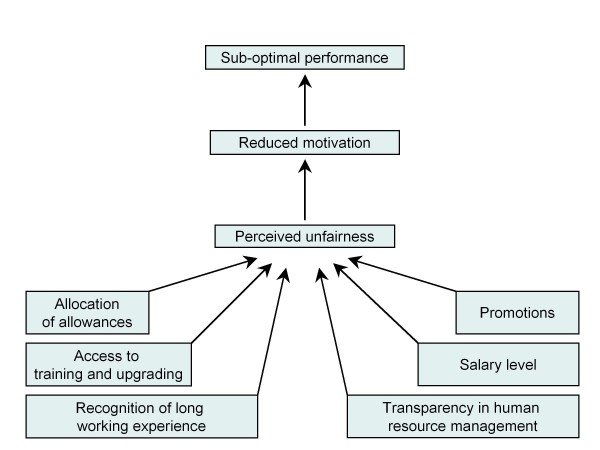
**The importance of health worker motivation**.

The health workers interviewed pointed out a range of examples of areas where they experienced unfairness as summed up in figure 1.

This study revealed substantial levels of frustration and even anger towards the employer, i.e. the hospital, district or national health authorities. At any workplace it can be expected that the relationship between the employer and the employees has an element of diverging interests. While the employer wants to maximise the ratio between the resources invested and the amount and quality of goods or services produced, the employees wish to maximise their return in terms of salary and other financial and non-financial incentives for work performed. This latent or manifest conflict commonly emerges in some form regardless of the availability of resources. The expectations of the employer and employees may be incompatible. The health sector management needs to strike a balance between employees' requests and what can be offered under the prevailing resource constraints.

### Perceptions of unfairness in a historical context

The level of frustration revealed in this study has emerged as surprisingly strong in light of the reforms in the health sector, in particular the significantly increased salary. We will however argue that it is impossible to understand the ways in which the perceptions of unfairness surfaced without discussing it in the light of the broad historical and political context particular to Tanzania. After the independence of Tanganyika in 1961, and the union with Zanzibar in 1964, Tanzania implemented free social services as a means to build the nation state [[42]:141]. There was a huge effort to provide basic services for the population through the African form of socialism, *Ujamaa*, implemented following the 1967 *Arusha Declaration *[43]. The political and social development was closely managed by the Tanganyika African National Union (TANU) and, after 1977, by the only political party, Chama Cha Mapinduzi (CCM). A basic welfare state was developed, and the expansion of the health sector was a result of a deliberate effort by the government, and health services were provided free of charge [[44]:60]. The success in providing welfare services in terms of health and education was a highly important achievement in the early development of the nation state. In the *Ujamaa *policies both peasants and government employees were attributed important roles in the development of the country. Health workers and teachers were seen as particularly important and were hailed in the nation-building project. The emphasis on health services and education was stressed by President Nyerere in the celebration of 10 years of independence in 1971 [[45]:292-301]. The fact that the government played an important role in the provision of welfare and in the close management of the economic sector was a very important aspect in creating expectations towards the state and its institutions. The expectations towards the state as a service provider naturally extend into the workplace relationship between the employer and the employee. One effect of these policies is that Tanzanians developed a set of expectations towards the government. In terms of management of the national economy, price control was first introduced in 1973 [[46]:36].

An enormous transformation has however taken place since the days of the post-independence optimism. In the 1980s, Tanzania suffered from large financial imbalances. Economic reforms were implemented through an economic recovery programme [[47]:31] and later through comprehensive structural adjustment programmes [48]. Economic hardship caused a deterioration of the public services, for example through a shortage of drugs and equipment in the health sector. A ban on new employment in the public sector worsened the situation. Another reason for the deterioration was a dramatic net reduction in the salary of civil servants. Stevens argues that by the late 1980s, "[i]n relative terms, the after-tax purchasing power of managerial, professional, and senior technical cadre salaries in the civil service was about one-twentieth of its value a decade and a half earlier" [[49]:68, see also [50]:6-7]. Mtatifikolo argues that for "the period 1975 to 1982 earnings of public sector employees declined at about 12 percent per annum in real terms" [[46]:35-36]. It is difficult to establish accurate data on salaries during this period, but there was without doubt a dramatic deterioration of salary level in relation to the cost of living. The availability of consumer goods was also limited, and the 1980s represented a period of severe problems for Tanzania as a country, and all the way down to employees and to the household level. The measures to improve the economy had a severe impact on the role of the state as a service provider as the population simply no longer received the free services they had become accustomed to [[51]:148-150, [52]:218, [53]:62, [54]]. Expectations towards the government generated during the early phase of the Tanzanian state have however continued to characterise the Tanzanian sentiment.

This context of optimism and the development of free services after independence followed by the severe resource constraints and the deterioration of salaries relative to the cost of living provide an important backdrop for the current findings of health workers' perceptions of their working conditions. The health workers interviewed expressed clear expectations towards the government as an employer. The cross-cutting issue was however that the government as employer was experienced as being incapable of fulfilling its responsibilities in caring for the employees. Some of the problems experienced were perceived to be the result of district-level decisions or central government policies. In relation to remuneration, the health workers interviewed readily acknowledged that the salary scheme is decided above district level, and thus placed the blame for the prevailing unfairness at the central government level.

Rousseau's concept of a 'psychological contract' may enhance our understanding of the dynamics at work. A psychological contract can be defined as "individual beliefs, shaped by the organization, regarding terms of an exchange agreement between individuals and their organization" [[55]:9]. The employee has a working relationship with a set of expectations concerning what the employment will yield in terms of financial benefits and other rewards. The psychological contract comprises the employee's perception of the employer's obligation to provide acceptable working conditions, and the employee will continuously evaluate the extent to which the psychological contract is fulfilled. The psychological contract is based on individual beliefs, but these beliefs are to a large extent shaped by the historical context. Whereas the formal working contract is legally binding on both parties, the psychological contract is held in the mind of the employee. A breach of the psychological contract may thus not be a violation of the formal contractual regulations of employment, but may cause an experience of dissatisfaction.

In our context, the health workers' complaints about salary level would imply a violation of the psychological contract, however as long as the salary is paid according to the official salary scale it is not a violation of the formal contract of employment. Delayed promotion is clearly a violation of the psychological contract, but may simultaneously be a violation of the formal contract if a fixed interval for promotion stated in the health worker's formal contract of employment is not adhered to. A district-specific payment of allowances may also be considered a violation of the psychological contract as it breaks with the principles of fairness associated with the state as an employer, but may not be a breach of the formal contract if the district-level health sector management has the authority to decide on the level and who should receive allowances. The importance of allowances has received increased focus and Ridde claims that health workers concern about maximising allowances may compromise the quality of health care [56].

The various cadres of health workers represent different levels in the hierarchical structure. The negative changes perceived are often accentuated and the positive changes are sometimes downplayed. The health workers interviewed accepted that national legislation and regulations place restrictions on district-level decision making. However, knowledge of relevant legislation proved to be restricted. The shortcomings in knowledge are partly caused by restrictions on the availability of information as in the case of health workers' complaints of little information on salary scales and allowances. However, the higher position in the health worker hierarchy, the more the health worker tended to be informed on how working conditions are regulated. The medical attendants demonstrated very little knowledge in this respect. The nursing staff and COs proved to have some knowledge, whereas the AMOs had relatively better knowledge about the rules and legislation regulating their working conditions. The present study has identified broad themes pertaining to perceived unfairness, but the need for acknowledging the variation within and between the cadres of health workers should be emphasised.

### The discourse on unfairness

The notion of unfairness with regard to the working conditions emerged as a cross-cutting issue throughout the FGDs and IDIs. The perception of unfairness took several forms, such as "we do not receive what we deserve in relation to the work we do" or "other health workers have better conditions than we have" but also "the government does not treat us well and does not care about our fate". Health workers' articulation of their experience of working conditions can be approached as a discourse. The concept of discourse refers to the expression of views and diverging interests [57]. The discourse we are concerned with here comprises health workers' interpretation of working conditions and their experiences of working in the public health sector. The repeated statements of working conditions being unfair can be seen as an attempt to voice the disappointment or frustration with the formal power relations between the employer and the employees.

In a very relevant anthropological work in Tanzania, Kamat has focused on the experiences of people who are caught in processes of rapid social transformation in the wake of structural adjustment programmes. In a study of discourses on health, Kamat points out that accounts of the past are reconstructed in current social contexts and reveals how people continuously relate the current situation with their perception and remembering of earlier times. Kamat refers to this phenomenon as the nostalgic discourse [[58]:364]. The perceived negative dimensions are often accentuated and the positive elements are commonly downplayed in this discourse. In the present study, the health workers interviewed who have a long working experience continuously draw upon the past when talking about the present, and their statements relating to working conditions may be interpreted as a nostalgic discourse. The claims of unfairness in the current working conditions, and in particular when they carry references to the past, could be considered as health workers' representations and not necessarily as historic facts as such.

### Diverse perceptions of unfairness

The perceived unfairness revealed in the present study is closely linked to the perception of justice at the workplace. A large body of literature relating to justice at the workplace exists. In these writings a basic distinction is made between *distributive *and *procedural justice*. Folger defines distributive justice as concerning the allocation of goods, for example salary and financial incentives, whereas procedural justice concerns "perceptions of the fairness of decision-making processes" [[59]:144]. From the data presented above, the health worker discourse on unfairness comprises a breach of both distributive and procedural justice. Health workers' evaluation of a breach of distributive justice is to some extent based on comparisons with the working conditions of other health workers.

Both horizontal and vertical comparisons take place at the workplace. Through vertical comparison health workers compare their own working conditions with other health workers at higher levels in the hierarchical structure. Nursing staff compare their own experience with what they know about the working conditions of the COs or AMOs. Many nurses argued that all cadres are important in running the health facility and find the differences unfair. One frequent claim was that the difference in allowances, for example the 'on-call allowance' being lower for nurses than for AMOs, was very unfair. Through horizontal comparison health workers compare their own work environment with that of colleagues in other districts or other health facilities within the same district. The health workers interviewed were very vocal about the differences in allowances between health workers within the same cadre. Differences in the level of allowances were clearly interpreted as a breach of distributive justice. Furthermore, there is extensive comparison with other government employees, for example teachers or staff at district council offices. Both the strike in 2005 and the complaints identified at district level tend to emanate from such comparisons.

Comparison becomes part of every formal system of employment and forms the grounds for negotiations about wages and working conditions, which in many countries is the domain of trade unions. The degree to which experiences of unfairness in working conditions emerge through comparison is high in this study. Another aspect of the comparison of working conditions relates to the perceived differences between the government sector and health facilities outside the government domain. Such comparisons involve a number of additional factors and nuances, e.g. pension schemes and work security, aspects that are too complex to be addressed in the present paper.

Another possible relevant point in the substantial expressions of unfairness is that the remuneration procedures pay less attention to long working experience and to the age and seniority of staff than is expected in this particular social and cultural context. Examples of health workers with long work experience and delayed promotions accentuate this dimension of perceived unfairness. The importance of local cultural traditions with great respect for seniority should not be underestimated. Kamat identified elders' perception of negative changes caused by macro-economic forces, and found that "elderly informants expressed public discontent with the political and economic system at large" [[58]:370]. The parallel in explaining deterioration as seen from the viewpoint of health workers who have experienced the changing economic conditions in Tanzania is notable.

### Increased opportunities to express dissatisfaction

Alongside the dramatic economic reforms, extensive political reforms also occurred during the 1990s. African one-party states came under increasing pressure to introduce democratic reforms, and in 1992 Tanzania gave up the one-party system and introduced a multi-party system, which came into action in the general election in 1995. Hydén describes the process in Tanzania as "creeping democratization" [[60]:152], distinguishing it from the more abrupt political changes seen in other African countries. Chama cha Mapinduzi (CCM) has managed to maintain its position as the ruling party, but Hydén also argues that "Tanzania is one of the better performers in Africa with respect to democratic governance" [[60]:143]. More important than the outcome of the multi-party elections was the increased space for debating the performance of the government and the national leadership. The emergence of opposition parties has created an arena for political debate that increasingly trickled down to local level. The media, in particular through the live TV broadcast of parliamentary debates as well as through the newspapers, has moreover increasingly challenged and criticised the government and has proven to the general public that the space for expressing criticism has significantly increased since the time of the single-party system. A large number of independent newspapers are published in Swahili and are therefore much more accessible to people than English language newspapers. The distribution of newspapers outside the urban centres is however limited and the cost of newspapers may be prohibitive to many Tanzanians, but there is no doubt that a remarkable improvement has occurred in terms of access to information and the opportunity to express critical views and opinions on politics and the performance of the government. This, together with the rapid increase in the use of cellular phones, means that information is spread quickly. As a consequence of the changes over the last two decades, both technical and political, Tanzanians have enjoyed both increased access to information and improved opportunities to express their dissatisfaction.

In 1999 Hydén claimed in broad and general terms that despite processes of democratisation "very few Tanzanians engage in collective action in order to promote or defend a particular idea or cause" [[60]:149], and that "[a]ssociational life in Tanzania is quite weak, even by African standards" [[60]:149]. He goes on to say that "Tanzanians still often tend to be deferential and prefer to keep quiet rather than to challenge authority in public" [[60]:152]. The transition from a history of top-down administration to multi-party politics, a market economy and harsh austerity measures has created an environment which may have appeared confusing in terms of where to direct sentiments and expressions of frustration. However, a decade later our findings indicate that the increased space for expressing dissatisfaction has been extensively used. As we have seen, the informants in this study expressed substantial dissatisfaction with the government as employer. We will argue that the striking frequency and strength of expressions of perceived unfairness and dissatisfaction in this study must be understood in light of such recent developments. However, despite the claims that the increased criticism has triggered restrictions in access to relevant information on salaries and allowances, the findings clearly show that health workers in the public health services are both vocal and articulate on issues pertaining to their working conditions and to the responsibility of the government as their employer.

### Methodological strengths and limitations

The number of IDIs and FGDs was considered to be sufficient for major themes to emerge over and over again, creating clear patterns of response. Data were collected after a considerable salary increase in the public health sector. At the same time new tools for human resource management were implemented and results-based financing was in the process of being introduced. We assume that such reforms to some extent could have coloured the responses given. A lack of transparency related to issues such as salaries, allowances and promotion policies led to difficulties in gaining access to reliable information. Interestingly, the lack of openness regarding this information simultaneously emerged as a key finding in the interviews and was a central reason for health workers' dissatisfaction.

Another limitation is related to the fact that Mbulu District may not be representative of rural districts in Tanzania, as a large voluntary agency hospital is situated in the district. There is however no indication that this has any particular influence on the working conditions in the public health sector.

Research partly carried out by a foreigner in a resource-constrained setting may however have had an impact on the data collected. In Mbulu District, and in Tanzania in general, many projects have been aid-based and implemented by foreigners. The researchers emphasised that there were no links between the present study and forthcoming interventions or policy initiatives. The opportunity to express dissatisfaction with the current state of working conditions may nonetheless have been somewhat exaggerated by the health workers during the interviews with a parallel downplaying of improvements in recent years.

## Conclusions

Thorough knowledge of health workers' experience of working conditions is needed to understand the determinants for motivation for work, and ultimately for the quality of health care. This study has focussed on health workers' experience of working conditions in a rural district in Tanzania. The theme emerging across all cadres of health workers was dissatisfaction with the working conditions. Salary level, promotion, recognition of work experience, allowances and upgrading opportunities, as well as human resource management were particularly emphasised by the study informants. The experience of not seeing the realisation of the expected working conditions clearly generates strong perceptions of unfairness.

We have argued that the discourse of unfairness pertaining to working conditions that was revealed in the present study must be understood in a specific historical and political context. We have thus made an attempt to situate the perceptions of unfairness within the dramatic shifts in Tanzanian modern history with an emphasis on Tanzania as a slowly maturing democracy. The soundness of the ongoing discourse is dependent upon the continued development of a free press and on an increased role of labour unions as constructive vehicles for expressing frustrations, as only through improved flows of information can health worker dissatisfaction and perceived unfairness be properly addressed. Recent historical developments in terms of openness emerge as promising for Tanzanian health workers, for their motivation to work, and by extension for the patients they are to serve.

## Competing interests

The authors declare that they have no competing interests.

## Authors' contributions

NGS planned and designed the study under supervision from AB. Data collection was carried out by NGS and DAM. The initial data analysis and development of the first drafts were carried out by NGS under supervision from AB. OBR and DAM contributed substantially to subsequent versions and critically revised the manuscript. All authors read and approved the final manuscript.

## Pre-publication history

The pre-publication history for this paper can be accessed here:

http://www.biomedcentral.com/1472-6963/11/34/prepub
